# Differences between work and leisure in temporal patterns of objectively measured physical activity among blue-collar workers

**DOI:** 10.1186/s12889-015-2339-4

**Published:** 2015-09-28

**Authors:** David M. Hallman, Svend Erik Mathiassen, Nidhi Gupta, Mette Korshøj, Andreas Holtermann

**Affiliations:** Centre for Musculoskeletal Research, Department of Occupational and Public Health Sciences, University of Gävle, Kungsbäcksvägen 47, SE 801 76 Gävle, Sweden; National Research Centre for the Working Environment, Copenhagen, Denmark

**Keywords:** Sitting, Accelerometry, Daily physical activity, Time pattern, Exposure variation analysis

## Abstract

**Background:**

Leisure time physical activity (LTPA) is generally associated with favorable cardiovascular health outcomes, while occupational physical activity (OPA) shows less clear, or even opposite, cardiovascular effects. This apparent paradox is not sufficiently understood, but differences in temporal patterns of OPA and LTPA have been suggested as one explanation. Our aim was to investigate the extent to which work and leisure (non-occupational time) differ in temporal activity patterns among blue-collar workers, and to assess the modification of these patterns by age and gender.

**Methods:**

This study was conducted on a cross-sectional sample of male (*n* = 108) and female (*n* = 83) blue-collar workers, aged between 21 and 65 years. Physical activity and sedentary behavior were assessed using accelerometers (Actigraph GT3X+) worn on the thigh and trunk for four consecutive days. Temporal patterns of OPA and LTPA were retrieved using Exposure Variation Analysis (EVA), and expressed in terms of percentage of work and leisure time spent in uninterrupted periods of different durations (<1 min, 1–5 min, 5–10 min, 10–30 min, 30–60 min and > 60 min) of sitting, standing, and walking. Repeated measures ANOVA and linear regression analyses were used to test a) possible differences between OPA and LTPA in selected EVA derivatives, and b) the modification of these differences by age and gender.

**Results:**

OPA showed a larger percentage time walking in brief (<5 min) periods [mean (SD): 33.4 % (12.2)], and less time in prolonged (>30 min) sitting [7.0 % (9.3)] than LTPA [walking 15.4 % (5.0); sitting 31.9 % (15.3)], even after adjustment for the difference between work and leisure in total time spent in each activity type. These marked differences in the temporal pattern of OPA and LTPA were modified by gender, but not age.

**Conclusion:**

We found that the temporal patterns of OPA and LTPA among blue-collar workers were markedly different even after adjustment for total physical activity time, and that this difference was modified by gender. We recommend using EVA derivatives in future studies striving to disentangle the apparent paradoxical cardiovascular effect of physical activity at work and during leisure.

## Background

Several studies have found that the relationship between physical activity (PA) and health differs depending on whether the activity occurs at work or during leisure [1, 2]. Moderate and high leisure time physical activity (LTPA) is generally associated with favorable health outcomes (e.g., reduced risk of cardiovascular disease and mortality), while no clear association or even an inverse relationship is observed for occupational physical activity (OPA) [2–6]. This apparent paradox still remains unexplained.

One possible explanation for the observed different effects of OPA and LTPA on health could be that temporal activity patterns differ between the two settings. OPA may be constrained to a particular type, duration, and intensity of PA, e.g., with limited opportunities for the worker to take breaks at discretion. In contrast, the individual is left to organize the contents and temporal structure of LTPA according personal preferences. Temporal patterns (i.e., variations across time) of different activity types, such as walking, standing and sitting, are considered important determinants for cardiovascular [7], metabolic [8] and musculoskeletal health outcomes [9], independent of the total exposure dose. Accordingly, at least 30 min per day of moderate PA accumulated in periods >10 min has been recommended for better health [10, 11]. Moreover, recent studies suggest that time spent in prolonged sitting (e.g., uninterrupted sitting periods >30 min) is particularly detrimental to health [12, 13], while sitting is not a health hazard to the same extent if accumulated from shorter periods. Thus, breaking up prolonged sitting by brief periods of walking or standing is associated with reduced resting blood pressure [14], enhanced endothelial function [15] and beneficial changes in biomarkers related to metabolism [8].

Epidemiological studies on health effects of OPA and LTPA have mainly relied on self-reported PA, and the instruments used for measuring PA have differed between work and leisure. Self-reports are prone to bias compared to objective methods for assessing OPA and LTPA [16, 17], and less reliable [18, 19], and they cannot be used to assess temporal activity patterns at any particular detail [20]. Thus, a trustworthy and detailed record of temporal patterns of PA needs to be based on an objective, valid and precise method for measuring PA across several days, such as accelerometry, accompanied by an appropriate analytical tool to retrieve the temporal structure of data.

Exposure Variation Analysis (EVA) [21] has been widely used to quantify temporal variation in long-term recordings of biomechanical exposures at work; most notably postures and muscle activity (e.g., [22–25]), but lately even daily PA, including sedentary behavior [26–28]. In the latter application, EVA splits up a time line of categorical PA data (expressed by type or intensity) into periods spent without interruption in the same PA category. Hence, using EVA, the temporal pattern of PA can be expressed as (proportions of) time spent in uninterrupted periods of different durations (e.g., <1 min, 1–5 min, 5–10 min, 10–30 min, 30–60 min and > 60 min) at different PA types (e.g., sitting, standing, running, cycling, walking).

Blue-collar work is associated with a substantial prevalence of musculoskeletal disorders and cardiovascular diseases [3–6, 29, 30]. Also, blue-collar workers, as opposed to, for instance, office workers, more obviously face the paradox mentioned above, i.e., that health effects of moderate-to-high levels of OPA and LTPA seem to be different. Although there have been efforts to objectively assess PA levels at work among blue-collar workers [31, 32], little is known about their temporal activity patterns. To our knowledge, no studies are available that compare these patterns in detail between work and leisure. Due to the dynamic nature of much blue-collar work, OPA may be distributed in relatively short periods of separate activities. In contrast, population studies suggest that leisure contains only few prolonged periods of (non-sedentary) PA and more periods of prolonged sitting than work. This PA pattern may be particularly pronounced among workers in blue-collar occupations due to their physically demanding work tasks [31]. Several studies suggest that PA differ depending on age and gender [33, 34], including the temporal activity pattern [26, 35]. Thus, age and gender may be modifiers of the potential differences in the patterns of PA between work and leisure.

Therefore, our aims were, 1) to document temporal patterns of objectively measured PA at work and during non-occupational time (henceforth referred to as leisure) in a cross-sectional sample of blue-collar workers, 2) to determine the extent to which these patterns differ between work and leisure, and 3) to assess the extent to which differences between work and leisure are modified by age and gender. We expected that the temporal distribution of time in any particular activity, including sitting, would differ between work and leisure, and that this would occur independently of total time in a particular PA type. Specifically, we hypothesized that standing and walking would to a larger extent occur as brief periods between other activities at work than during leisure, while prolonged sitting periods would occur more during leisure than during work.

## Methods

### Study population and design

The present study was conducted on a cross-sectional sample of male (*N* = 108) and female (*N* = 83) blue-collar workers from the ‘New method for Objective Measurements of physical Activity in Daily living (NOMAD)’ study in Denmark. Data were collected from October 2011 to April 2012. Danish surveys and registers were used to select seven occupational groups with a high prevalence of musculoskeletal disorders and with varying exposures to OPA (i.e., workers in the health service sector, assembly workers, cleaners, construction workers, manufacturing workers, garbage collectors, and mobile plant operators). Workers were then recruited by convenience from different workplaces, primarily through contact with trade unions or safety representatives. Workplaces were eligible if workers were allowed to participate in the study during paid working hours.

Individuals were allowed into the study if they performed blue-collar work as their main occupation for at least 20 h per week, and if they were between 18 and 65 years of age. Workers were excluded if they declined to sign an informed consent to participate, reported to predominantly perform white-collar work, were pregnant, were absent from work due to sickness on the day of testing, or reported skin allergy to adhesives.

In total 358 blue-collar workers were offered participation, out of which 259 volunteered to participate and 223 filled out a questionnaire and used the accelerometers. Out of the 223, 10 workers were excluded as they reported their working hours to be less than four hours per day, and 22 workers were excluded because not even one working day with valid objective measurements was available. Thus, 191 workers were included in further examination of OPA and LTPA. The study was approved by the regional Ethics Committee in Copenhagen, Denmark (journal number H-2-2011-047) and conducted in accordance with the Helsinki declaration.

### Procedure

Prior to data collection, all workers were invited to information meetings where the objective, procedure, and requirements of the study were explained. Workers declaring an interest in taking part in the study completed a screening questionnaire containing general information about demographic variables. Each participating worker were instructed to wear accelerometers for collecting PA for 24 h per day over four consecutive days, with research staff visiting the worker at the workplace on the first and last day. On the first day, the worker (a) underwent anthropometric measurements, (b) was equipped with accelerometers for objective measurement of OPA and LTPA, and a written diary, and (c) completed a computer-based questionnaire (results presented elsewhere [36]).

The worker was instructed to perform a reference measurement in upright standing for 15 s each day, to report the times of those reference measurements as well as non-wear time in the diary, and even to note times when getting up in the morning, starting and ending work, and going to bed. The worker was allowed to remove the accelerometers if they caused itching or any kind of discomfort such as disturbed sleep. After completing the four measurement days, the worker returned the objective measurement devices, and the accelerometer data were downloaded to a computer by the research staff.

### Assessment of age and gender

Age was determined from the workers’ Danish civil registration numbers, while gender was assessed using self-report.

### Objective assessment of physical activity

Physical activity was measured continuously using two accelerometers (Actigraph GT3X, ActiGraph LLC, Florida, USA) placed on the thigh and trunk using double sided adhesive (3 M, Hair-Set) and medical tape (Fixomull, BSN medical), as previously described [36, 37]. The Actigraph is a small, water resistant device (19x34x45mm, weight 19 g), which records, samples and stores tri-axial acceleration data at a frequency of 30 Hz with a dynamic range of ± 6G, and a 12 bit precision.

The Actigraph was initialized for recording and downloading of data using the manufacturer’s software (Actilife Software version 5.5, ActiGraph LLC, Pensacola, FL, USA). The accelerometer data were further processed and analyzed using a custom-made MATLAB based software, Acti4 (The National Research Centre for the Working Environment, Copenhagen, Denmark and BAuA, Berlin, Germany), which determines the type and duration of different activities and body postures with a high sensitivity and specificity [38–40]. In this software, accelerometer data were low-pass filtered using a 5 Hz 4th order Butterworth filter and then split up into 2 s sequences with 50 % overlap. Afterwards, using the individual’s reference measurement, the occurrence of different PA types (i.e., sitting, standing, walking, running and cycling) was identified from the accelerometer outputs using algorithms presented previously [37, 38]. Walking periods interrupted by brief (<30 s) sequences of standing, moving, running or cycling where merged if the total duration of the walking period exceeded 10 min. Non-wear was identified when, (a) the software detected a period longer than 90 min with zero acceleration counts, or (b) the participant reported non-wear time, or (c) artefacts or missing data were detected by visual inspection.

On average, the data collection period included 2.0 working days containing both work and leisure (i.e., non-occupational time). As only working days were addressed in this study, non-working days were excluded from the analyses, as were periods of sleep and non-wear, as well as periods not coded in the diary. The total non-wear time in the population was 1 % for the thigh and 3 % for the trunk accelerometer. A working day was considered valid for further analysis only if it contained objective measurements for at least four hours of work and >75 % of the average (across days) reported working time. Also, a day was accepted only if it comprised at least four hours of leisure, and >75 % of the average (across days) reported leisure time. Prior to further analysis on OPA and LTPA, each included working day was split into periods of “work”, defined as self-reported time spent working, and periods of “leisure”, defined as the waking hours not spent working, as described above.

### Exposure Variation Analysis of physical activity (EVA)

For each measurement day, the time-line of the processed accelerometer signal was analyzed using EVA, identifying the occurrence of uninterrupted periods of different durations (i.e., <1 min, 1–5 min, 5–10 min, 10–30 min, 30–60 min and > 60 min) in each PA type (i.e., sitting, standing, running, cycling and walking). For each worker, average time spent in different EVA cells (e.g., sitting without interruption for 1–5 min) was expressed in minutes per day (i.e., total minutes in a particular EVA cell divided by the number of measured working days) and as percentages (minutes/day in a particular EVA cell divided by the average measured minutes/day). This was done separately for OPA and LTPA. Referring to PA recommendations from the American College of Sports Medicine and the American Heart Association [10], and the 2008 Physical Activity Guidelines for Americans [11] we then derived four selected derivatives from the EVA matrix according to Straker et al. [27], i.e., “*prolonged sitting*” (time spent in uninterrupted sitting periods >30 min), “*brief bursts (BB) standing*” (time spent in <5 min standing periods) and “*BB walking*” (time spent in <5 min walking periods), and “*walking >10 min*” (time spent in >10 min walking periods). These metrics comply with recommendations based on biomedical evidence [10, 11, 15, 27, 41–43] and operationalize the characteristics of OPA and LTPA addressed in the driving hypotheses of our study.

### Statistical analyses

Descriptive data are presented as mean and standard deviation (SD) between workers, or frequencies. Time spent in different PA categories were averaged across days and expressed in percentages or minutes. All statistical analyses were carried out using the software SPSS, version 22 (IBM, US). The level of significance (α) was set at p < .05. Variables were visually inspected for normal distribution, and no deviations were identified that preclude the use of parametric procedures as described below.

The difference in temporal activity patterns between work and leisure as expressed by the selected EVA derivatives (i.e., *prolonged sitting, BB standing, BB walking* and *walking >10 min*) was tested using repeated measures ANOVA; time period (two levels: work, leisure) was treated as a within-subject factor. To determine whether differences between patterns of OPA and LTPA occurred independently of total PA time, repeated measures ANCOVA was used to adjust for the difference in total PA time between work and leisure (ΔtotalPA, i.e., LTPA subtracted from OPA; ANCOVA). Partial eta squared (η^2^) was used as a measure of effect size. ANOVAs were also constructed with time period (two levels: work, leisure) as a within subject factor and gender as a between subject factor to investigate the main effect of gender and the interaction (gender × time) on each EVA derivative.

Linear regression models were used to identify a possible association between each EVA derivative and the total time in each activity type, both during work and leisure. Associations between the EVA derivatives in OPA and LTPA were expressed using Pearson correlation coefficients. For each EVA derivative, a multiple regression model was developed to retrieve the possible association of age and gender with the difference between work and leisure in that EVA derivative (i.e., the LTPA result subtracted from the OPA result).

## Results

### Descriptive statistics

**Fig. 1:**
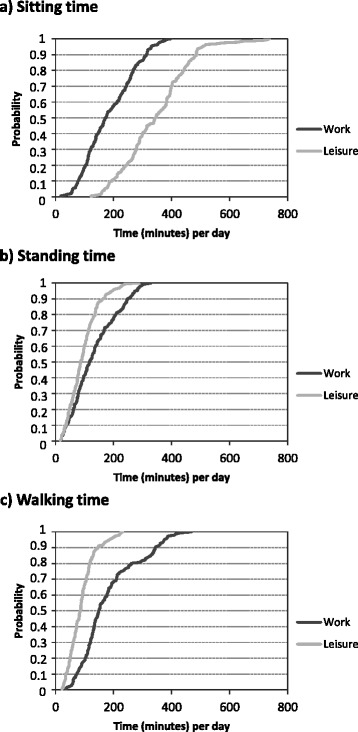
Cumulative probability distributions of sitting (**a**), standing (**b**), and walking (**c**) during work and leisure. *N* = 191 in all diagrams

Accelerometer data was available for 191 male (*N* = 108) and female (*N* = 83) blue-collar workers, with, on average, 2.0 (SD 0.9) included working days. The average age of the workers was 45 (SD 9.5) years. The average duration of work and leisure was 8.4 h/day (SD 2.5) and 8.9 h/day (SD 2.7), respectively. The studied occupations were clearly dominated by either males or females, but genders did not differ in age, body mass index or accelerometer wear-time (Table 1). Total time spent in different activities was markedly different between work and leisure (Table 2 and Fig. 1). Compared with OPA, LTPA was characterized by a larger proportion of time spent sitting, and less proportion of time spent standing and walking. Time spent running and cycling was higher during leisure than work, but very small in both cases. Since very little time was spent in running and cycling, these variables are reported for descriptive purposes but not analyzed further (Table 2). Fig. 1 shows the cumulative distributions of sitting, standing and walking for work and leisure.

**Table 1:** Descriptive data for male (*N* = 108) and female (*N* = 83) blue-collar workers

	Males	Females
Age (years); m (SD)	43.8 (9.5)	46.9 (9.3)
Body mass index (kg/m^2^); m (SD)	26.8 (4.4)	25.8 (5.9)
Measured working days per worker; m (SD)	2.0 (0.9)	1.9 (0.8)
Wear-time work (hours/day); m (SD)	8.4 (2.6)	8.3 (2.1)
Wear-time leisure (hours/day); m (SD)	9.2 (2.9)	8.6 (2.2)
Occupation, N		
Health service	0	14
Assemblers	4	27
Cleaners	5	26
Construction	38	0
Manufacturing	31	16
Garbage collectors	19	0
Mobile plant operators	10	0
Other occupation	1	0

**Table 2:** Total time spent in different physical activities during work and leisure. Mean (m) and standard deviation (SD) of time per day, in terms of Minutes and percent, spent in different physical activities at work and leisure among blue-collar workers (*N* = 191), and the number (N) of workers with at least one detected period in each specific activity

	Work	Leisure	Work	Leisure	Work	Leisure
			m (SD)	m (SD)	m (SD)	m (SD)
	N	N	Minutes	Minutes	%	%
Sit	191	191	187 (88)	346 (115)	39.4 (19.2)	65.3 (11.8)
Stand	191	191	133 (78)	95 (51)	25.1 (11.0)	17.5 (7.4)
Walk	191	191	182 (100)	90 (45)	35.4 (13.8)	16.5 (5.8)
Run	4	17	0.0 (0.2)	0.5 (2.4)	0.0 (0.1)	0.1 (0.6)
Cycle	41	45	0.4 (1.4)	3.4 (10.7)	0.1 (0.3)	0.6 (1.7)

### Exposure variation analysis of OPA and LTPA

**Fig. 2:**
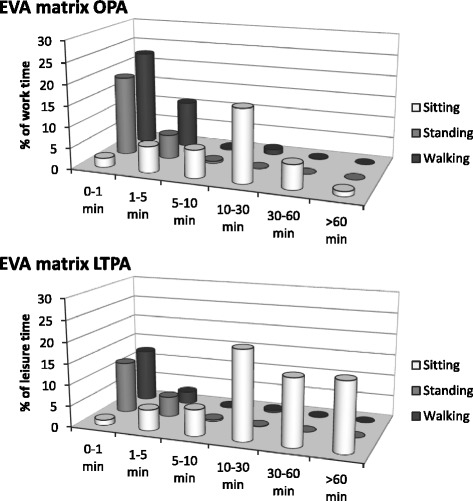
Exposure variation analysis (EVA) for occupational (OPA) and leisure time physical activity (LTPA) averaged across all blue-collar workers (N = 191). The x-axis shows categories of uninterrupted periods (ranging from 0–1 to >60 min), the y-axis shows the three different activity types (sitting, standing and walking), and the z-axis shows accumulated time (% time averaged across days). Time spent in brief bursts of standing, i.e., *BB standing*, and walking, i.e., *BB walking*, was calculated by adding time in periods of 0–1 and 1–5 min of standing and walking, respectively. Time spent in walking periods >10 min, i.e., *walking >10 min* was calculated by adding time in walking periods of 10–30, 30–60 and > 60 min. *Prolonged sitting* was obtained by adding time in sitting periods of 30–60 and > 60 min

Figure 2 shows the average EVA for OPA and LTPA in the study population. Temporal patterns of OPA and LTPA were significantly different for all EVA derivatives (Table 3, ANOVA, all p < .05). The difference between OPA and LTPA was mainly due to a larger proportion of time spent in *prolonged sitting* (>30 min periods) during leisure (on average 31.9 % (SD 15.3)) compared to work (on average 7.0 % (SD 9.3)). Time spent in *BB standing* (<5 min periods), *BB walking* (<5 min periods) and *walking >10 min* was less during leisure than during work. Despite substantial time spent in walking (Table 2, Fig. 1), very little walking time, i.e., on average 1.5 % (SD 4.6) at work, and 0.8 % (SD 2.2) during leisure, was accumulated in periods >10 min duration. Differences between OPA and LTPA in EVA derivatives were also tested after adjusting for the difference between work and leisure in total time spent in each activity (Table 3). OPA and LTPA remained significantly different for all EVA derivatives besides *BB standing*, although the effect sizes became smaller with adjustment for total time (e.g., for *prolonged sitting*, partial *η*^*2*^ was reduced from .68 to .29).

**Table 3:** Differences between work and leisure in temporal patterns of physical activity. Repeated measures ANOVA for tests of differences between occupational and leisure time physical activity in selected EVA derivatives (% time in prolonged sitting, BB standing, BB walking, and walking > 10 min)

		ANOVA			
		df	F	*p*	η^2^
Unadjusted model (ANOVA)	Prolonged sitting (>30 min)	1,190	396	**<.01**	.68
	BB standing (<5 min)	1,190	65	**<.01**	.25
	BB walking (<5 min)	1,190	344	**<.01**	.65
	Walking >10 min	1,190	3.8	**.05**	.02
Adjusted model (ANCOVA^a^)	Prolonged sitting (>30 min)	1,190	77.7	**<.01**	.29
	BB standing (<5 min)	1,190	2.7	.10	.01
	BB walking (<5 min)	1,190	12.5	**.01**	.06
	Walking >10 min	1,190	11.2	**.01**	.06

*BB* brief bursts, *EVA* Exposure variation analysis

^a^ANCOVA adjusted for the difference (Δ) between work and leisure in total time for each specific activity type

Significant values (*p* < .05) are bold faced

### Associations between total activity time and EVA derivatives

**Fig. 3:**
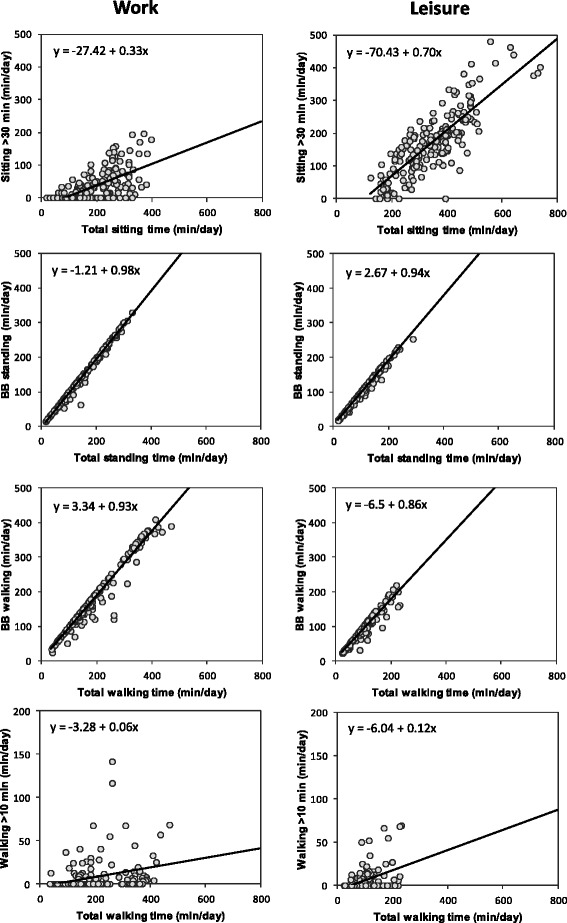
Associations between the four selected EVA derivatives (*rows, y-axes*) and total accumulated time (*x-axes*) in different activity types at work (*left*) and during leisure (*right*). The linear regression equation is shown in each plot; all *N* = 191

The EVA derivatives were positively associated with total time spent in each activity type, and these associations appeared different for work and leisure (Fig. 3). For OPA, clear positive associations (Fig. 3) were found for *BB standing* (r^2^ = .99) and *BB walking* (r^2^ = .96) with total time standing and walking, respectively. *Prolonged sitting* and *walking >10 min* were also positively associated with total time, but the residual variance in EVA metrics at any particular total time were considerably larger than for the *BB* metrics (r^2^ = .40 and r^2^ = .10, respectively). For LTPA, all EVA derivatives showed positive associations with total time spent in the corresponding activity type, and as for work, residual variance was smaller for the *BB* metrics (*BB standing*, r^2^ = .99; *BB walking*, r^2^ = .93; *walking >10 min*, r^2^ = .20; *prolonged sitting*, r^2^ = .69). There were no clear associations between values during work and leisure for any EVA derivative (*r* ranged between -.05 and .08; all with *p* > .05).

### Effect of age and gender

Table 4 shows EVA derivatives for males and females separately. Main effects of gender indicated that males spent more time in *prolonged sitting* and *walking >10 min,* and less time in *BB standing* than females. Interaction effects (gender × time) were found for *BB standing*, *BB walking*, and *walking >10 min* indicating that males and females changed their PA patterns from work to leisure to different extents. Gender, but not age, was significantly associated with the difference (work vs leisure) in the EVA derivatives *BB standing*, *BB walking*, and *walking >10 min*, as suggested using multiple regression (Table 5). Thus, female workers showed larger decreases from work to leisure in *BB standing*, and smaller reductions in walking, compared to males.

**Table 4:** Temporal patterns of physical activity during work and leisure among males and females. EVA derivatives (percent time; mean (SD)) among males (n = 108) and females (n = 83). ANOVA shows p-values for the main effect of gender and the interaction gender × time

	Work		Leisure		Main effect			Interaction	
	Males	Females	Males	Females	df	F	*p*	F	*p*
Prolonged sitting (>30 min)	8.2 (10.2)	5.6 (7.7)	34.8 (15.1)	28.2 (14.9)	1,189	12.2	**<.01**	2.5	.11
BB standing (<5 min)	21.1 (9.5)	29.0 (10.9)	15.4 (6.3)	18.9 (7.2)	1,189	47.4	**<.01**	5.4	**.02**
BB walking (<5 min)	34.5 (11.7)	32.1 (12.8)	14.8 (5.2)	16.2 (4.5)	1,189	0.2	.63	3.9	.05
Walking >10 min	2.5 (5.9)	0.3 (1.0)	0.7 (2.0)	0.9 (2.4)	1,189	7.7	**<.01**	9.8	**<.01**

ANOVA was constructed with gender as a between subjects factor and time period (work vs leisure) as a within subject factor. Significant (*p* < .05) main effects of gender are shown in bold

*BB* brief bursts (<5 min), *EVA* exposure variation analysis

**Table 5:** Effects of age and gender on temporal patterns of physical activity. Results of multiple regression analyses using age and gender as independent variables and the difference (Δ) between work and leisure in EVA derivatives (time (%) in *prolonged sitting*, *BB standing*, *BB walking*, and *walking > 10 min*) as dependent variables. B-coefficients indicate the effect of an increase of 1 year of age (Age), and of being female (Gender)

			Age			Gender		
EVA derivative	Intercept	R^2^	B	β	p	B	β	*p*
Prolonged sitting (>30 min)	−30.6	.01	0.00	0.00	1.0	4.00	0.12	.12
BB standing (<5 min)	−1.9	.03	0.08	0.06	.44	4.15	0.16	**.03**
BB walking (<5 min)	2.2	.03	0.13	0.09	.21	−4.23	−0.16	**.03**
Walking >10 min	18.3	.06	0.05	0.08	.25	−2.47	−0.24	**<.01**

*BB* brief bursts (<5 min), *EVA* exposure variation analysis

Significant values (*p* <.05) are bold faced

## Discussion

The current study documented temporal patterns of different types of objectively measured physical activity (PA) at work and during leisure among blue-collar workers, determined the extent to which these patterns differed between work and leisure, and investigated whether they were modified by age and gender.

### Temporal activity patterns and compliance with guidelines

Despite that total time spent walking was considerable (i.e., on average 182 min per day for OPA, and 90 min per day for LTPA), almost all of this time (i.e., 96 % during work and 95 % during leisure) was accumulated in short uninterrupted periods (i.e., less than 5 min). This suggests that very few of the workers reached the health recommendation of performing at least 30 min per day, five days a week, of moderate-intensity PA accumulated in periods exceeding 10 min [10, 11]. This limited compliance with common PA guidelines agrees with previous accelerometry-based studies [32, 44]. The extent of walking may therefore not be sufficient to lead to positive health effects. However, cardiometabolic health benefits have been observed even from performing PA in shorter bouts (i.e., < 10 min) [8, 45], particularly if they interrupt prolonged sedentariness [14, 15, 43]. Worth of note, however, guidelines are largely based on studies using self-reported PA, and therefore our results based on objective measurements cannot be directly compared to the suggested limits.

Sitting time was, in contrast to walking, accumulated in longer periods (Fig. 2), even during work. There is growing evidence for an association between prolonged sitting and deleterious health outcomes, independently of the extent of moderate-to-vigorous PA [46, 47]. Prolonged sitting is known to be common in white-collar occupations dominated by office-based tasks [26, 28, 48]. Our study shows that prolonged sitting at work (i.e., periods >30 min) also occurs among blue-collar workers, which is consistent with data indicating a general reduction in the intensity of OPA over the last decades [49]. Although the health consequences of sitting during work have not yet been established among blue-collar workers [12], our results suggest that some workers would be exposed to sitting to an extent associated with health risks. In addition, the workers spent a considerable proportion (on average 32 %) of their time during leisure in prolonged sitting. This suggests that interventions aiming at interrupting prolonged sitting at work and during leisure could be relevant to this population, and that investigations of how to accomplish such changes are needed not only among workers in what is usually regarded as sedentary occupations, such as office work [50].

### Different temporal patterns of OPA and LTPA

We found that temporal patterns of PA, as assessed objectively using accelerometry, were markedly different between work and leisure. OPA showed a larger time spent in *BB walking* and *BB standing* than LTPA, while LTPA showed a markedly larger occurrence of *prolonged sitting* compared to OPA. Total time spent in each activity type also differed substantially between work and leisure (Table 2), and it explained a substantial proportion of variance in several of the EVA derivatives (Fig. 3). We therefore adjusted for differences in total time in our statistical models, but still found that the temporal pattern of OPA and LTPA differed significantly; with the marginal exception of *BB standing*. This is also apparent when inspecting the residual dispersion around the regression lines in Fig. 3: a particular total time in sitting, standing and walking may be distributed differently for different workers, most notably with respect to the occurrence of *prolonged sitting *and *walking >10 min*. Thus, EVA derivatives contain unique information about the temporal structure of PA among blue-collar workers that cannot be predicted from knowing only the total time in PA. Also, a particular total time in PA was, on average, distributed differently for work and leisure, as illustrated by the different slopes of the associations between total time and EVA derivatives in work and leisure (Fig. 3). Again, the difference was most pronounced for *prolonged sitting* and *walking >10 min*. Thus, we claim that EVA provides unique and important information beyond that available and/or predictable from data only on total time spent in PA categories.

There is abundant evidence for positive health effects of increased LTPA (e.g., [51]), while the health effects of OPA are much more ambiguous, with studies even showing negative health effects of increasing OPA [4, 6, 31, 52]. For instance, in a prospective study among nurses, high levels of OPA was associated with an increased risk of ischemic heart disease, and this effect was strongest among nurses who were sedentary during leisure time [6]. Several studies have found contrasting effects of OPA and LTPA on cardiovascular disease (CVD) risk. A recent meta-analysis based on 23 prospective studies found that high levels of LTPA are associated with a moderately reduced risk of CVD, while moderate and high levels of OPA slightly increased the risk of CVD [2]. In that study, the authors propose that different temporal structures of PA at work and during leisure could be a likely explanation of these opposing effects. Still, few studies have investigated and compared detailed temporal patterns of OPA and LTPA [28], and studies addressing blue-collar workers are particularly scarce. Thus, our study provide novel results in this respect, and we recommend future studies of the health effects of temporal patterns in PA to use EVA derivatives based on objective measurements of PA. We would particularly encourage studies to investigate whether the influence of the temporal PA pattern on health differs between OPA and LTPA. Moreover, future research should also investigate whether health effects of different PA patterns, if any, persist after accounting for psychosocial factors and stress, as these factors may obviously differ between work and leisure.

### Effects of gender and age

A study of call-center employees found sit/stand patterns at work to differ between males and females, with females sitting to a larger extent in long, uninterrupted periods [26]. In contrast, among the present blue-collar workers, males spent more time in prolonged sitting at work than females (Table 4). We also found that males spent less time in short periods of standing than females, and accumulated more walking time in long (>10 min) periods at work.

One explanation to this gender effect could be that males and females were employed in different occupations, which require physical work of different temporal structures. However, since the seven occupations included in the current study were dominated by either males or females (for details, see [36]), it was not possible to address occupation and gender as independent effect modifiers in the statistical models. This said, we observed different temporal patterns between genders even for LTPA (Table 4), which suggests that the present findings were not exclusively due to an effect of occupation.

For some of the investigated EVA derivatives, we found that males and females differed in the relationship of temporal activity patterns at work versus leisure (Table 4). The regression models adjusting for age (Table 5) showed significant associations between gender and the difference in PA between work and leisure (Δwork-leisure), i.e., female workers showed a larger decrease from work to leisure in *BB standing* than males, and smaller reductions in *BB walking* and *walking >10 min*. This corroborates that gender is an effect modifier for the temporal patterns of PA, including differences between OPA and LTPA, and we encourage future studies to consider gender stratified analyses when investigating the possible health effects of PA patterns at work and during leisure. Age was weakly associated with several EVA derivatives (Table 5), but none of the associations were statistically significant in this population. Thus, we could not confirm age to be an effect modifier for the difference in temporal patterns between OPA and LTPA.

### Methodological discussion

A major strength of the current study is the use of accelerometry to assess OPA and LTPA across several working days. Self-reported measures of PA are less valid and reliable than objectively measured data [18, 20, 39], and cannot be recommended for determining temporal patterns of PA. Furthermore, the combination of two accelerometers allowed us to quantify sitting time, which is not possible using only one accelerometer. The Acti4 software, which we used in the current study, has shown good sensitivity and specificity in detection of different PA types from the accelerometer signal [40]. Thus, we consider our results to be accurate.

Reflecting the growing interest in temporal patterns of PA, including sedentary behavior at work and during leisure, several recent papers have proposed methods for retrieving the temporal structure of objectively measured PA [26–28, 48, 53–55]. The most simple of these methods count the number of separate sitting periods in a day, and calculate the mean duration of periods longer than a specified threshold [48, 55]. Going in more detail, the paper by Toomingas et al. [26] addresses the time-line of sitting and non-sitting, and suggests a number of variables expressing, e.g., frequencies of transitions, occurrence of prolonged sitting periods, and compliance with guidelines for regular breaks from sitting. Chastin and Granat [53] also consider sitting vs. non-sitting. On the basis of a mathematical approximation of the structure of the exposure time-line, they propose the so-called GINI index to measure, on a scale from 0 to 1, the extent to which the total cumulated time in sitting is composed of short or long periods (“bouts”). While the methods in the four papers by Ryan et al., Stephens et al., Toomingas et al., and Chastin and Granat all operate on a dichotomous exposure time-line, the approach presented by Paraschiv-Ionescu [54] is conceptually similar to the present EVA procedure in classifying PA into several different types, and reporting the occurrence of uninterrupted periods in (some of) these PA types throughout a day. The time-line of activity types, illustrated as a “bar-code”, is then analyzed in terms of its basic descriptive statistics, and its dynamic properties, e.g., entropy. The latter analyses reflect the extent to which different activity types occur in deterministic, “self-similar” temporal structures. Finally, Parry and Straker [28] and Straker et al. [27] use an approach based on EVA that is essentially equivalent to the one used in the present paper, even if the set-up of activity categories is different.

While epidemiological and experimental studies indicate the importance of temporal patterns of PA for health [14, 15, 56], most of the approaches referred above of how to operationalize temporal patterns, including the descriptive metrics they produce, have, at this stage, only a hypothetical bearing on health. The EVA approach used in the present study to determine temporal patterns of PA has the advantage of being useful for any discretionary set-up of two or more activity categories. It provides transparent and intuitively interpretable data, and it can be adapted to reflect compliance with PA recommendations. Worth of note, however, EVA cannot capture the real-time succession of periods, such as, for instance, whether a long period in sitting is always followed by a long period of non-sitting [9]. Some of the cited papers offer ideas of how to address such properties [26, 54], and Paraschiv-Ionescu et al. [57] have presented an overview of analyses of the temporal dynamics of activity patterns for clinical use.

Since our sample was selected by convenience, it may not be considered representative to a general population of blue-collar workers. Also, the current sample contained different numbers of male and female workers, and the seven included occupations were highly gender-stratified. Thus, the observed gender differences in temporal PA patterns need to be replicated in studies that, if possible, address populations with a more balanced structure of males and females between and within occupations. Finally, our study did not discriminate between different sub-domains (e.g., house holding, gardening and transportation) of LTPA. Future studies should examine the temporal structure of objectively measured PA in different sub-domains since they may entail different effects on health and wellbeing [58].

## Conclusion

We found the temporal patterns of occupational and leisure-time physical activity (PA) among blue-collar workers to be markedly different even at similar levels of total PA time. This difference was modified by gender. Our results stimulate further research into the significance of temporal PA patterns to health, and we encourage using EVA derivatives based on long-term objective measurements of PA in this research.

## Abbreviations

EVA: Exposure variation analysis
OPA: Occupational physical activity
LTPA: Leisure time physical activity
PA: Physical activity
